# Thinking Differently About Same Day Access: Qualitative Study of Primary Care Staff Experiences in England and Denmark

**DOI:** 10.1111/hex.70921

**Published:** 2026-10-23

**Authors:** Tina Drud Due, Sue Ziebland, Thomas Bo Drivsholm, Anna Dowrick

**Affiliations:** ^1^ Department of Public Health University of Copenhagen Copenhagen Denmark; ^2^ Nuffield Department of Primary Care Health Sciences University of Oxford Oxford United Kingdom

**Keywords:** access, demand, focussed ethnography, general practice, primary care, qualitative study, same day access

## Abstract

**Context:**

Rising demand for GP services alongside limited change in supply is placing unprecedented pressure on primary healthcare systems in many countries, including the UK and Denmark. The 2026 English GP contract requires practices to provide same day access to all patients considered to have an urgent need.

**Objectives:**

To study primary care staff reflections on their experiences of prioritising same day access through different approaches, which their practices had chosen and adapted over months and years.

**Design:**

A subset of three case studies (two in England, one in Denmark) from a larger focussed ethnography. These practices were selected for the sub‐study because they all prioritised same day access but had developed different approaches. We draw on formal and informal interviews with staff and observations in the practices.

**Results:**

The different rationales for introducing same day access in these three practices had been to reduce long waiting times, to tackle non‐attendance at pre‐booked appointments and to help address limited GP capacity. Systems were maintained by weekly and daily tinkering with appointment capacity, by managing the balance of access and continuity, understanding where exceptions were important for different patient groups and, in the English case studies but not in Denmark, by channelling patients to allied health care professionals. In all cases, consideration was given to the trade‐off for patients between urgency and continuity. Staff drew on their own experiences to caution against entirely open appointment booking systems. Staff in all three sub‐study practices had, based on their experiences, started to think differently about how best to manage same day access. Our findings, comparing the English and Danish practices, raise the counter‐intuitive possibility that, rather than opening the floodgates, making it easier for patients to access GP appointments when needed might help moderate total demand.

**Conclusions:**

These findings matter in relation to the 2026 GP contract, which has specified that 90% of appointments deemed ‘urgent’ by the practice must be seen on the same day. When dealing with seemingly overwhelming demand, it is tempting to introduce channels and diversions to protect supply, or even to question whether the demand is justified. Our findings suggest a pertinent metaphor: it is now recognised in flood prevention that the introduction of man‐made channels has exacerbated the problem of flash flooding, while the use of open, naturally managed flood plains is beneficial. This metaphor may help practices to understand the consequences of their response to the new requirement for same day access.

**Patient and Public Contribution:**

The wider ethnographic study was guided from inception to completion by a PPI co‐applicant and eight PPI representatives who, between them, represented a diversity of lived experience. In relation to this article, the PPI co‐applicant reviewed practice summaries and provided sense‐checking of our ideas and interpretations.

## 1 Introduction

### 1.1 Crisis of Access in Primary Care

Access to primary care is a cornerstone of effective healthcare systems, in many countries serving as the initial entry point for patients to have their health needs met [1]. Timely and equitable access to primary care services is essential not only for individual health outcomes but also for public health and healthcare efficiency at large [2]. It is increasingly understood that primary care services must be designed with a diversity of users in mind, embedding accessibility [3] and trauma‐informed care as standard practice [4].

The 2026 GP contract for England includes, among other changes, requirements for same day access for 90% of patients judged to have an urgent need for care and for practices to identify patients for whom continuity of care is particularly important (NHS England, 2026) [5]. While the intention to improve access has been welcomed, there are concerns about the likely impact on an already stretched primary healthcare service facing unprecedented pressure. Factors driving these pressures include an ageing population, an increase in chronic diseases, heightened patient expectations, rising costs of healthcare delivery, and the transfer of tasks traditionally managed in secondary care to general practice [6, 7]. A range of national and local initiatives have been introduced to address this gap. A recent systematic review categorised these innovations into two groups [8]. First, interventions that seek to modify routes by which patients obtain appointments, including introducing telephone triage [9], online consultation [10] and redirecting towards non‐GP professionals [11]. Second, interventions that seek to change appointment capacity, for example, limiting advance booking, extending hours or shortening appointment times [8, 12]. To date, there has been no ‘silver bullet’ identified among these adaptations, and fears abound that practices will be ‘flooded’ with patients if barriers to access are lowered.

In light of the 2026 GP contract in England, this paper examines primary care staff reflections on their experience of different approaches to same day access, which their practices had chosen, developed and adapted over months and years. The settings, Denmark and England, have similar publicly funded primary care systems that are struggling to ensure that those most in need of care get timely access [13, 14]. The resulting strain on general practice has led to significant challenges for patients' access, manifesting in longer wait times and reduced continuity of care [15]. These issues, further fuelled by adverse media attention, have been recognised to undermine healthcare outcomes and diminish both patient and staff satisfaction [15, 16, 17].

Influential policy reports in both countries have framed enhanced workforce capacity as the solution to access problems [15, 18]. In Denmark, reforms are taking place that aim to redistribute health care funding, emphasising community‐based leadership in improving access to primary care services [19]. In the United Kingdom, the 10‐year health plan similarly prioritises community‐based service provision, with improved digital access and distribution of routes to accessing care and advice across a broader range of primary care professions [20]. The 2026 GP contract sharpens the focus on same day access for urgent care needs alongside targeted continuity of care for specific patients [5].

### 1.2 Reframing Access as Flow

Discussions of access typically emphasise the point of entrance into primary care services, and foreground efficiency in how patients get appointments. Studies approaching service design from the broader perspective of patient flow look beyond initial access to consider movement through services. They highlight that understanding the specific needs of patient populations and flexibility within service capacity are of equal importance in service design as establishing an efficient process of entry [21, 22, 23]. Voorhees et al describe access as the human fit between the needs and abilities of people in a population and the abilities and capacities of people in the healthcare workforce [21]. Service designs which neglect to address these factors can increase demand instead of improving the flow of patients through appointments.

A range of models have been introduced across time to address access to appointments with different consequences. Evaluation of these models has found that multiplicity of access systems results in greater complexity, and that different patient groups value different approaches, trading‐off preferences regarding speed of access, choice of appointment times and choice of professionals [24, 25, 26, 27]. In this sub‐study (for the full study findings see [27]), we focus on practices that have prioritised same‐day access. Among the few studies of patient flow in same‐day access in primary care, a Swedish study reported a reduction in demand when a same day, self‐triage ‘See and treat’ initiative was introduced within primary care [28]. The authors concluded that the observed ‘reduction in visits and telephone contacts could indicate that, somewhat paradoxically, increasing access to primary care physicians does not “open the floodgates” nor increase demand'.

In this paper, we build on the above literature, exploring how primary care staff reflect on their experiences of same day access models. Three practices (two in England and one in Denmark) were selected from a focussed ethnography of a wider variety of access systems [25]. In other work, we have explored the perspectives of patients on different access systems, illuminating how layering and complexity of access approaches can confuse and frustrate patients [25, 29]. Building on these studies, in this contribution, we explore staff experiences of implementing and adapting their provision of same day access, examining the initial rationale and their reflections on how improved patient flow might be achieved in this context.

## 2 Methods

The wider ethnographic study of access in General Practice involved fieldwork in eight English practices, and one Danish practice, all of which had introduced and adapted various ways of managing appointments [25]. Here, we focus on a subset of three case study practices which had all prioritised same day access yet had, over months and years, developed notably different approaches [30] (see Table 1).

**TABLE 1:** Sub‐study practices prioritising same day access.

**Practice reference**	**Location**	**Example of changes made to access system**	**Rationale for practice approach to same day access**	**Ethnographic data collected**
E ‐ England	Market town, midlands	Newly established acute care team set up to deal with same‐day access. This included clinical telephone triage leading to some face‐to‐face appointments. Pre‐booked appointments with own GP to maintain continuity.	Balancing continuity with same‐day access for urgent issues.	Interviews: GPs: 5 Receptionists: 5 Other: Care coordinators 2 Hours of observation: 64 [Drawn from a potential sample of 12 GPs, 9 reception staff, 10 nurses, 4 care coordinators, 1 practice manager]
H ‐ England	Urban, northern	Appointments same‐day only. Patients can email practice via an e‐consultation system. Increased use of allied health professionals. To protect appointments for same day GP access reception (care navigator) staff encourage patients to see nurses, physiotherapists, physician assistants or pharmacy instead of GP.	Had been a high ‘did not attend’ rate for pre‐booked appointments. Shortage of GP sessions created pressure to prioritise same day GP appointments for those in most clinical need.	Interviews: GPs 2 Receptionists 4 Practice manager 1 Care coordinator 1 Hours of observation: 45 [Drawn from a potential sample of 9 GPs, 8 receptionists, 5 nurses, 2 care coordinators, one practice manager].
Denmark	Urban, large city	Pre‐booked appointments (only chronic disease check‐ups, pregnancy, and child examinations) can be arranged online, by phone, or during a prior visit. All other appointments regardless of urgency are scheduled on the same day and arranged by phone in the morning. Patients can email GP directly to get results from blood tests and ask specific, short questions.	Difficulty meeting demand, long waiting time, staff overload.	Interviews: 9 GPs 4 Receptionists 2 Practice manager 1 Nurse 1 Care coordinator 1 (Drawn from a potential sample of 4 GPs, 3 nurses, 2 receptionists and 1 care coordinator) Hours of observation: 15

We observed reception spaces and held informal discussions with staff during the summer and winter of 2023 in the English practices and in 2022 in Denmark. Researchers wrote detailed fieldnotes from observations, which informed topic guides for interviews. Qualitative semi‐structured interviews were conducted with GPs, nurses, reception staff, care navigators and allied health professionals. We concentrated on interviewing staff who could reflect on how and why their practice's approach to same day access had changed, providing an unusually rich source of data for this sub‐study. All participants gave written informed consent and gave their time to the study voluntarily.

The interviews were collected using a similar topic guide across the practices and covered the access system for that practice, how it has changed since being introduced, perceived impact on patients and any unintended consequences. The majority of interviews were face‐to‐face in the practice, with a few conducted on the telephone. All of the formal interviews were audio‐recorded, transcribed and checked for accuracy by the research team.

We were interested in the adjustments that staff made within their systems, drawing on the concept of ‘tinkering’ [28] to characterise the work of recalibrating patient flow. Mol et al [31] argue that good care is characterised by ‘persistent tinkering in a world full of complex ambivalence and shifting tension’. Here, tinkering refers to a continuous, experimental process of finding local, practical solutions to same day access. We were also interested in what staff considered to be the limits of ‘tinkering’ in order for systems to function [25, 31].

The interviews were conducted in Danish in Denmark and English in England. As a first step, the English and Danish researchers coded their own interviews and fieldnotes from the subset of practices in the original language [32]. At a data workshop with all four authors, held in Copenhagen in April 2024, field notes and interview excerpts were explored using the OSOP mind‐mapping approach, facilitated by the Danish team members doing a rough translation of extracts of data to supplement the English data [33]. This approach uses constant comparison to ensure that all of the relevant data contribute fully to the analysis. Coded data included staff talking about adjustments and modifications to local needs, daily fluctuations and continuity, priority patients and unintended consequences of prioritising same day access. This led to the three themes for this paper: rationale for the same day approach to managing patient flow; tinkering and flexibility in the system; drawing on experience to think differently about demand. These themes were developed through iterative discussion among the co‐authors. Subsequent refinements to the line of argument were elaborated by sharing and modifying drafts, which included searching for examples of deviant cases that contradicted the main findings.

The researchers all had English as either a first or additional language and met for analysis sessions in person and online. Selected interview extracts to be used in the paper were formally translated from the Danish by the bilingual researcher in preparation for this article. The thematic analysis is illustrated with quotes from interviews. The study received ethical approval from the Health Research Authority (HRA) and Health and Care Research Wales (HCRW) reference: 22/SC/0333.

## 3 Results

### 3.1 Rationale for Introducing Their Same Day Approach to Managing Patient Flow

English Practice H had transitioned to a same day only appointment system in response to identifying a problematic ‘did not attend’ (DNA) rate. Practice H serves a population experiencing high deprivation, and staff understood why patients might forget to attend or cancel a pre‐booked appointment, but this had caused problems given pressures on GP availability.

It's quite logical that a lot of people make appointments and forget about them and they don't show up, so then, say for example if that's five wasted a month, that's five appointments that other people could have had.

(Practice H—receptionist)

In English practice E one of the GPs described the need to meet ‘on the day demand’ as part of the rationale for introducing triage by clinical staff. A GP here recognised inefficiencies associated with pre‐booked appointments and the importance of clinical triage, noting that consultations with patients who had waited for 4 weeks often ‘didn't need to be seen or could be dealt with in a split second’ (Practice E—GP).

The Danish practice had changed to a same day access system due to difficulties for GPs in managing workload and demand, which had resulted in concerns about waits of several weeks for patients wanting non‐acute appointments. The shift to same day appointments was for everything other than chronic care check‐ups and preventive services, regardless of the perceived urgency of the patient's issue. Staff in the Danish practice reported that the patients appreciated the system, which had resulted in dramatically decreased waiting times and led to fewer no‐shows and complaints. It had also increased staff job satisfaction.

In the old days, people would call in and I knew I had a free appointment in 3 weeks, and I knew I had one in 4 weeks. Then someone would call saying they had a [non‐urgent] rash, and we'd give them the 3‐week slot. Then someone else would call with a bad knee, so we'd have to give them the 4‐week slot. And then we'd think, ‘Damn, that should've been the other way around’, and we'd spend an incredible amount of time on that. There were also daily urgent slots to fit people in, and we spent so much time on it—it was our responsibility to find time for the patient. Today, they call in and tell us what they need, and we help them assess not only how soon they need to be seen but also how they can be seen most effectively and how we can make the best use of our resources.

(Danish practice—Nurse)

Across the three sub‐set practices, ensuring sufficient supply of appointments to meet same‐day demand required establishing different systems of triage. In English Practices E and H, maintaining sufficient same day appointments with GPs meant ‘navigating’ some patients to other clinicians in the team, or other services in the community. English practice H placed particular emphasis on increasing efficient use of other clinicians in the practice (physiotherapist, GP assistant, nurse) and had a formal relationship with their local community pharmacy, which helped patients with minor illnesses. This could help protect their scarce GP appointments for those most in need.

What we're trying to do is work smarter with what we have. So we're using the care navigation, we've got quite a few allied services. Like we have a minor eye conditions service that we can refer patients to. So if someone booked an eye appointment then online they might be able to use that service, same with the physio. We also have the community pharmacy scheme, so sort of coughs, colds, viral illnesses, verrucas, warts all those sorts of things that can be comfortably dealt with by the pharmacist.

(Practice H—GP)

The Danish practice used telephone triage to assess need. Practice phone lines were only open for a couple of hours in the morning, and calls were answered by the whole practice team. They would determine which issues could be handled on the phone, advise patients if they thought they should ‘wait and see’, and allocate the day's appointments between GPs and GPs in training, nurse consultations and video consultations, or direct patients to online text‐message‐based communications with the GP (e.g., for test results and brief questions).

The agreement with the patient is that if they need an appointment, they will get one. The person in charge stays until there's nothing left to do—that's how it is! But sometimes, you could say there can be a discrepancy between what the patients feel is needed and what we actually believe is needed.

(Danish practice—GP)

There are a few things that can be handled over the phone, but if the patient has an issue and wants it looked at, then it's not really up to me to decide whether the patient should come in or not. It's kind of the patient's decision. Unless I can reassure the patient and say, ‘Well, you know what, this is my assessment, you don't need to come in’ then the patient might say, ‘Oh, phew,’ because there are also many who call just for advice.

(Danish practice—Nurse)

Staff working in the Danish practice thought that the shift to same‐day appointments had increased trust between staff and patients, in that patients did not book appointments ‘unnecessarily’ and were often happy to ‘wait and see’ for a few days, knowing they would be seen by a doctor on whichever day they called.

Patients show more understanding, and I also think we find that there are some issues that can wait when they know they can call tomorrow. They can call later in the day and maybe get an appointment, or they can call tomorrow and get one. If the stuffy nose they have hasn't improved, it calms people down because it's not like in the old days when you had to wait 3 weeks, or even 4 or 6 weeks. They know they can get an appointment; they just have to call early the next day. And they know they can see the doctor they want if they just call early enough.

(Danish practice—Nurse)

### 3.2 Tinkering and Flexibility With Same Day Access

Across all three practices, staff discussed the balance of keeping their system intact while tinkering when exceptions were needed. Tinkering took place on a day‐to‐day basis, as well as in longer‐term planning.

An important trade‐off was continuity with a patient's own GP versus the urgency of addressing their issue that day. Staff in Practice E were keen to maintain continuity of care alongside a mix of same day (urgent) and pre‐booked (non‐urgent) appointments. This involved everyday work, tweaking and adjusting appointment capacity. A GP at Practice E reflected on the daily tinkering with appointment capacity, where new slots could be opened later in the day, and how this related to maintaining a responsive balance of continuity and urgency.

We're trying to get the balance between efficiency of actually dealing with the problem there and then, versus continuity or just whether that's pushing the problem onto somebody else, we're just trying to put our discretion of judgement to try and get that right. But if it's a minor problem where continuity doesn't matter then it should get sorted out on the day by nurse or a doctor in that way.

(Practice E—GP)

Another GP from Practice E discussed the challenge of prioritising between continuity and urgency in complex cases.

There's a group for where continuity is appropriate, and there's a group of people for whom access is the most important thing and that's appropriate. If you can devise a system that mops up most of those, the group in the middle which are the harder ones, for where access is quite important with their own doctor, but also access is more, is important and they might not realise it. And that sort of grey area is the one where we really have to develop, refine our systems better because they're ones who potentially, either are made to wait too long or are getting sort of too many series of transactions on an urgent basis without someone really stepping back going ‘wait a minute what's going on? This is the fourth time you've been in with that’.

(Practice E—GP)

The commitment to maintaining continuity of care with the patient's own GP was recognised as valuable in all three sub‐study practices, though Practice E staff said they would prioritise continuity if a patient could wait. From 2026, the English GP contract requires practices to identify the patients for whom continuity of care is needed. This was not the case when we conducted our fieldwork, although in both English practices we witnessed informal flexibility by reception staff where there were known additional care needs. This chimes with the Danish practice, where continuity was considered especially important for some groups of patients but could be traded‐off against same day access for others. If a patient wanted a same‐day appointment and their GP was not available, they were given the option of seeing a different GP or calling back another day.

You could say we're not necessarily interested in everyone having a designated doctor. In a way, it's something assigned to those who need it. For example, I've sometimes told young people who have mentioned this, ‘You're a young, healthy person; it's not that important,’ and they often see that point. So, in that sense. But in principle, it has nothing to do with this system at all. If it were important to them, that would be perfectly fine. They can simply choose to be assigned an appointment with (name of doctor). If there are no more slots with (name of the GP), they can call tomorrow or the day after.

(Danish practice—GP)

Practice H and the Danish practice staff said that people were not allowed to book ahead, although both allowed some flexibility. While we were observing Practice H, the reception team discussed a concerning situation where an elderly patient who appeared to have cognitive difficulties came to the desk asking to see the GP. All of that day's appointments had already been allocated. The reception staff member was worried that the patient might struggle to remember to call back the next day and proposed to the team that they might hold a small number of appointments to be bookable for the next days. These would not be advertised to patients but could be used at the receptionist's discretion. Those at the team meeting welcomed this as a sensible workaround which met the needs of these patients while also maintaining the integrity of the existing same‐day appointment system.

The Danish practice made exceptions for some vulnerable patients and a few with work schedule constraints. While the system relied on everyone adhering to the agreed structure, staff in the Danish practice argued that the system only works because exceptions are sometimes made. Each day, a GP, nurse and receptionist would work together to manage the flow of appointments, enabling capacity for a few buffer slots for an extra consultation in the afternoon if patients called after the same day appointments had run out.

You have to stay true to the system otherwise it will collapse. So, we have to—well, we remind each other from time to time to hold each other accountable for that. You're not supposed to break it, so to speak. But it has helped a bit that we've set up these buffer times for ourselves, as it makes it easier to manage.

(Danish practice—GP)

The approach used by the Danish practice allowed patients to decide whether they needed a same‐day appointment, whereas the two English practices used care navigators, reception and clinical staff to decide who qualified for a same day appointment and with whom.

In interviews, we discussed staff views about online systems which would allow patients to log on and select their own appointment slots from a diary. Practice H and the Danish practice had each trialled an online booking system for GP appointments. While staff recognised that the approach was popular with patients, it was considered a step too far and both practices had abandoned the approach, as this receptionist explained.

They would book anything and everything; you cannot care navigate if patients are booking their own appointments. You can't go to them and say, ‘Actually, you'd be better off going there’. So we felt the online booking system was… was wasting a lot of clinicians’ time with irrelevant things that patients really didn't need to speak to a GP about.

(Practice H—receptionist)

In the Danish practice, an early iteration of their same‐day appointment system had an online booking option. Staff said that they preferred the phone triage system. Even though this added a layer of work, it maintained some control over appointment booking. A completely open system where patients were responsible for selecting their own appointment slots in an online diary was not deemed appropriate by any of the staff we interviewed in the sub‐study practices.

### 3.3 Thinking Differently About Managing Demand

Pressures on general practice in both countries had increased, and all staff recognised that making changes to their access systems must be done alongside improving public understanding of what was reasonable to expect from a constrained primary care service.

I think the patients that have been here (local town) years don't realise how quickly the population is growing compared to the resources that there are, maybe. So I think the older generation kind of think that they're less likely to call and will come to the desk and try and get an appointment at the drop of the hat. Think the doctor will see them there and then if they wait around because they've known them for ages. That's not the case.

(Practice E, Receptionist)

As noted above, different systems placed different responsibilities on patients or staff to decide whether a same‐day appointment was needed. Of the three case studies, the patients registered with the Danish practice appeared to have the greatest latitude (and also the greatest responsibility) in determining their need for a same day appointment, as the following quotes illustrate.

Well, I think the good thing about it is that some of the responsibility is given back to the patients. It's a hundred times, a thousand times easier to triage when you have appointments available the same day. It allows you to say, ‘Well, I can't help you with an appointment today if you can't make any of the three times I've offered you, so you'll need to call on another day.’ It's up to you to prioritize how important this is for you.

(Danish practice—Nurse)

I feel that they need to assess for themselves whether they think it's important enough to see a doctor. If people feel that it's not important enough to take a day off for, then I don't need to see them either. We have these video consultations, and they can do that from work, which they do quite a lot. They call in with small issues when they're at the office, and many things can be resolved that way. But if they need to come in, they have to prioritise it.

(Danish practice—GP)

Our final observation about managing demand differently may appear counter‐intuitive. The Danish practice has made it much easier to book an appointment, when needed, on the day that the patient decides it is needed. Rather than leading to overwhelm, staff told us that the practice appears to have reduced overall demand. This chimes with the observation from Savage et al [22] and also with an observation from an English GP at Practice E that too much energy is dedicated to trying to channel patients away from the GP, while many of their concerns could be dealt with quickly on a single call with an experienced clinician. Staff in the Danish practice suggested that because on‐the‐day access to appointments was now guaranteed, the overall demand had fallen. Reasons for this were thought to include a greater willingness to ‘wait and see’ for a day or two, as well as the observation that patients were less likely to ‘squirrel’ pre‐booked appointments to use even when their original issue had resolved.

But I've experienced elsewhere [other practices] that people come in and say, ‘I actually booked for my knee; it's gotten much better, but now that I have the appointment, I wanted to ask about…’ and I find that somewhat absurd [….] its nice that with the current system people bring in what's on their minds and that it doesn't turn into some kind of strange performance.

(Danish practice—GP)

## 4 Discussion

Despite years of research and policy initiatives aiming to improve access to primary care, there is no consensus on how to develop a successful national approach. In this analysis we explored localised responses to the challenge of providing same day access. Three case study sites, in high‐income European nations with publicly funded health systems, were grappling with the familiar challenge of meeting high demand for appointments in general practice in the context of resource and workforce constraints. We interviewed and observed staff who reflected on their experience of different same day access approaches, which their practices had chosen and adapted over months and years. Their views are particularly illuminating in relation to the 2026 English GP contract, which requires same day response to needs deemed urgent by the practice. This study offers two insights about staff reflections on using same day approaches that may be helpful to practices. (1) Same day access solutions developed in response to local context and ‘tinkered with’ over time may prove more effective than national ‘grand blueprint’ solutions and (2) thinking differently about access and easing the barriers to access might (counterintuitively) even reduce demand.

### 4.1 Trade‐Offs When Tinkering With Locally Developed Same Day Access

In this sub‐study all three same day access systems were developed in response to local context. Staff adapted according to fluctuations in predicted demand and staffing, to attend to the needs of particular patient groups, and to balance the trade‐off between continuity of care and same day access. While none reported adverse events, staff were mindful that any approach to access might disadvantage some groups of patients. This reinforces the argument that acknowledging and supporting responsive locally developed access system are a better approach for shaping patient flow [21, 22, 23]. To ensure the most effective use of patient, practice and health care resources, there was consensus among staff that some form of triage or involvement in appointment booking was essential.

We observed variation between practices in the role that staff wanted patients to have in deciding if a same‐day appointment was needed. In the Danish practice, patients were guaranteed an appointment on the day they sought it. Patients were responsible for determining the right time to seek help and for organising their day to accommodate the appointment. In the English practices, there were extra layers of work involved for primary care staff in establishing whether the need was sufficiently urgent for a same day appointment and perhaps diverting the patient to another clinician.

### 4.2 Could Demand be Reduced by Easing the Barriers to Same Day Access?

When dealing with seemingly overwhelming demand, it is tempting to introduce barriers and diversions to protect the supply or to question whether the demand is justified. This can have unintended consequences. For example, if an appointment with a GP is hard to obtain and the patient has to wait several days, or even weeks, then the appointment also acquires a rarity value. Perceived as a precious commodity, it will probably not be relinquished even if it is no longer needed for the original purpose. On the contrary, making an appointment easy to achieve may reassure the patient that they can ‘wait and see’, secure in the knowledge that an appointment will be there when it is needed. This aligns with the finding that easing direct patient access to a primary care ‘See and Treat’ same day clinic resulted in an overall reduction (definitely not a flood) of visits and appointments [28]. Returning to the concept of patient flow [22, 23], there is little evidence to suggest that patient flow can be slowed down and made more manageable by creating filters, diversions and limiting access.

Our findings suggest a pertinent metaphor: it is now recognised in flood prevention that the introduction of man‐made channels has exacerbated the problem of flash flooding, while the use of open flood plains, alongside the reintroduction of beavers, has been beneficial. Initiatives that attempt to manage the natural course of rivers through straightening the banks and creating narrow channels exacerbate flooding by building up pressure. Recent years have seen the (re)introduction of more successful flood management initiatives, where the relaxation of attempts to channel and confine streams is combined with the reintroduction of naturally dam‐building beavers [34]. Beavers continuously monitor, adjust and repair dams. This metaphor may help illuminate for practices how to respond to requirements for same day access. The analogy connects Kriendler's [22] contention that there are rarely simple or elegant solutions to the complex problem of patient flow with Mol et al's [31] emphasis on tinkering with care practices. Instead of trying to establish fixed routes for controlling and channelling patient flow away from GP appointments, the most effective solutions are likely to be local, layered and responsive, requiring regular recalibration.

This finding adds nuance to national policies on increasing same day access to primary care, which emphasise increasing workforce capacity and digital access routes [15, 18, 19, 20, 21]. While increasing capacity is indeed important for the long‐term sustainability of primary care systems, primary care staff will continue to develop and adapt locally responsive access systems [27].

### 4.3 Strength and Weakness

While the data were collected some time before its introduction, the findings from this sub‐study have particular significance in relation to the 2026 GP contract. A strength of this study is in the depth of data collection. Experienced qualitative social scientists generated rich, reflective case studies based on formal and informal interviews and observations with field notes in the practices. The three sub‐study sites, in high‐income European nations with publicly funded health systems, were grappling with the familiar challenge of meeting high demand for appointments in general practice in the context of resource and workforce constraints. These three case studies were selected because they had chosen and adapted different approaches, over months and years, to address same day access. The reflections of the staff in these practices are particularly illuminating because they are grounded in the experience of locally instigated changes and adaptations over time.

Tinkering is normal, and probably laudable, in the context of access innovations in primary care. However, attempts by individual practices or wider organisations to learn from their adjustments is hampered by the lack of an agreed design and set of (routine) data to indicate ‘what works’. The development of a core data set which practices could use to assess their own initiatives and share findings with others could support more informed access initiatives alongside pragmatic national and international comparisons. This could be an important area for future research. Without such indicators, there is a danger of tinkering in the dark, risking premature or delayed abandonment of innovative approaches, including initiatives to address the new GP contract requirements to provide same day access in urgent cases.

## Author Contributions

**Tina Drud Due:** conceptualisation, investigation, data curation, formal analysis, writing – original draft, writing – review and editing. **Sue Ziebland:** conceptualisation, investigation, data curation, formal analysis, writing – original draft, writing – review and editing. **Thomas Bo Drivsholm:** writing – review and editing. **Anna Dowrick:** formal analysis, writing – original draft, writing – review and editing.

## Ethics Statement

Ethical approval for the UK‐focused ethnography was provided by the South Central Hampshire A Research Ethics Committee on 10th October 2022 (reference 22/SC/0333). The Danish case study was not classified as a research study needing ethics approval under the Danish Act on Research Ethics ‐ Review of Health Research Projects Section 2. Confirmation that the study could be initiated without approval from The Committees on Health Research Ethics for the Capital Region of Denmark was obtained on 3rd August 2022.

## Consent

All participants provided written and verbal informed consent prior to participating.

## Conflicts of Interest

The authors declare no conflicts of interest.

## Data Availability

The data sets generated during the current study are available to bona fide researchers for secondary analysis upon reasonable request. All data requests should be submitted to the lead investigator for consideration. No data will be shared earlier than 12 months after the end of the study.
